# Evaluating Group Housing Strategies for the *Ex-Situ* Conservation of Harlequin Frogs (*Atelopus* spp.) Using Behavioral and Physiological Indicators

**DOI:** 10.1371/journal.pone.0090218

**Published:** 2014-02-25

**Authors:** Shawna J. Cikanek, Simon Nockold, Janine L. Brown, James W. Carpenter, Angie Estrada, Jorge Guerrel, Katharine Hope, Roberto Ibáñez, Sarah B. Putman, Brian Gratwicke

**Affiliations:** 1 Department of Clinical Sciences, College of Veterinary Medicine, Kansas State University, Manhattan, Kansas, United States of America; 2 Panama Amphibian Rescue and Conservation Project, Smithsonian Tropical Research Institute, Gamboa, Republic of Panama; 3 Smithsonian Conservation Biology Institute, National Zoological Park, Front Royal, Virginia, United States of America; CNRS, France

## Abstract

We have established *ex situ* assurance colonies of two endangered Panamanian harlequin frogs, *Atelopus certus* and *Atelopus glyphus*, but observed that males fought with each other when housed as a group. Housing frogs individually eliminated this problem, but created space constraints. To evaluate the potential stress effects from aggressive interactions when grouping frogs, we housed male frogs in replicated groups of one, two, and eight. We measured aggressive behavioral interactions and fecal glucocorticoid metabolite (GC) concentrations as indicators of stress in each tank. In both small and large groups, frogs initially interacted aggressively, but aggressive interactions and fecal GCs declined significantly after the first 2 weeks of being housed together, reaching the lowest levels by week 4. We conclude that aggressive interactions in same-sex groups of captive *Atelopus* may initially cause stress, but the frogs become habituated within a few weeks and they can safely be housed in same-sex groups for longer periods of time.

## Introduction

Amphibian biodiversity is being lost at an unprecedented rate [1] prompting the creation of *ex-situ* assurance colonies of endangered species as part of a global ‘Amphibian Ark’ effort coordinated through the IUCN [2]. *Atelopus* species are a high priority for rescue and assurance populations because of their susceptibility to the invasive fungal pathogen, *Batrachochytrium dendrobatidis* (*Bd*), which has devastated naïve upland amphibian communities throughout Panama [3], [4]. The Panama Amphibian Rescue and Conservation Project was created in response to these *Bd*-related declines and consists of two *ex-situ* facilities in Panama that house populations of amphibians; the El Valle Amphibian Conservation Center (EVACC) and the Smithsonian Tropical Research Institute's Gamboa Amphibian Research Center (Gamboa ARC). Collectively, these facilities house five of the six Panamanian *Atelopus* species: *A. zeteki* Dunn, 1933; *A. varius* (Lichtenstein & Martens, 1856); *A. limosus* Ibáñez, Jaramillo & Solís, 1995; *A. certus* Barbour, 1923; and *A. glyphus* Dunn, 1931. The sixth known harlequin frog species, *A. chiriquiensis* Shreve, 1936, has not been observed since 1996 and may be extinct [5].

The Panama Amphibian Rescue Project aims to create assurance colonies of 20 endangered amphibian species and to grow the captive population of each species to a minimum effective population size of 500 individuals [6], [7]. However, space is a major limiting factor for *ex situ* programs, and housing frogs in groups would greatly expand our capacity to meet our population management goals. The AZA species survival plan for *A. zeteki* manages about 2,000 captive *Atelopus* and frogs are regularly housed in same-sex groups to limit the extended periods of amplexus observed in opposite sex groups (K. Murphy, pers comm). Many of these frogs have been raised in captivity in group-housing situations, and therefore acclimated to group conditions, but it was unclear how readily our wild-caught *Atelopus* would acclimate to group housing.

To evaluate the levels of stress associated with different housing scenarios for captive *Atelopus,* we developed behavioral and physiological indicators to quantify stress. Types of *Atelopus* calls include advertisement, release, territorial, and courtship with the most common being advertisement [8]. *Atelopus* males also use visual signals, such as semaphore foot-raising, to signal antagonistic behavior [9], and their territorial behavior has been well studied [10], [11], allowing us to compile an ethogram to observe and document aggressive interactions as a behavioral indicator of stress.

Glucocorticoids are a group of steroid hormones that can be used as indicators to evaluate stress, health status, and disease in many species including amphibians [12], [13]. Glucocorticoid release is the last step of a hormonal cascade that begins in the brain to help an animal react to a stressor [14], [15]. An animal's internal response to stress involves the activation of the hypothalamic-pituitary-adrenal axis (HPA), and the release of cortisol or corticosterone from the adrenal cortex [16]. These GCs can be measured in urine, feces, plasma, and blood [17]. Blood analyses are the most common, but not always the most practical because of the potential stress of sample collection that would be too invasive to employ on small-bodied, endangered *Atelopus,* e.g. [17], [18], [19]. Methods to evaluate corticosterone in amphibian urine have been developed, validated and tested on several species [20], [21], [22], but handling and manipulating individuals to collect the urine sample is still somewhat invasive, especially with smaller, more delicate frogs. By contrast, fecal pellets are readily collected from captive frogs without disturbance, so we adapted and validated existing fecal GC tests as a non-invasive approach to evaluate physiological stress responses in frogs. The goal of this study was to use behavioral and physiological indicators to determine if wild-caught *A. certus* and *A. glyphus* could be maintained in same-sex groups without compromising animal welfare.

## Methods

### Husbandry

Facilities to house wild-caught amphibians from Central America were established in Gamboa, Panama. Collections of from the Darien region were made with permission from the Autoridad Nacional del Ambiente (ANAM) permits SE/A-130-10 and SE/A-42-11. The Animal Care and Use Committee of the Smithsonian National Zoological Park approved the project(#09–31). A total of 44 *A. certus* and 22 *A. glyphus* were housed individually in small plastic cages measuring 28×19×16.5 cm for at least 1 year before the start of this 5-week study. Cages were misted daily and enriched with native plant leaves (*Philodendron* spp.) and damp brown paper towels. Tanks were kept on metal racks with fluorescent overhead lighting for 12 hours per day and cleaned twice per week. At the start of the experiment, frogs were removed from the plastic cages and placed in large, numbered glass tanks (size 25×53×38 cm) with automated misting systems lightly spraying the tank interiors for 5 minutes every 2 hours. Cages had false bottoms installed (plastic egg crate covered in 0.5 mm screen mesh), keeping frogs (and fecal pellets) out of contact with any dirty water that may have pooled on the tank bottom. Ultraviolet-emitting lights supplemented the 12-hour overhead fluorescent lights for eight 45-minute intervals per day. Each tank was furnished with two potted plants (*Philodendron* spp.), rocks and a water basin. Frogs were randomly assigned to one of three treatments consisting of identical tanks housing one, two, or eight male *Atelopus* frogs, respectively. Each treatment was replicated 6 times in a completely randomized design. *A. glyphus* males (mean Snout Vent Length (SVL)  = 37 mm SD +/−1.8 mm, 3.76 g SD +/−0.53 g) were used in 2 full replicates, and *A. certus* males (mean SVL  = 32.3 mm SD +/−1.6 mm, 2.65 g SD +/−0.39 g) were used for the remaining 4 replicates. Black, opaque dividers were placed between tanks to limit visual cues from neighbors. Frogs were fed ad libitum with small crickets (*Acheta domesticus*) or fruit flies (*Drosophila melanogaster and D. hydei*) dusted with calcium or vitamin supplements 4 times per week. Frogs were weighed and measured (SVL) at the start and end of the experiment. We examined overall weight loss or gain as a measure of body condition, expressed as the relative change in mass as a percentage of the starting weight. We created cage cards using photographs of the unique pattern of dark spots clearly visible on their white ventral side to distinguish between individuals.

### Behavior

**Figure 1:**
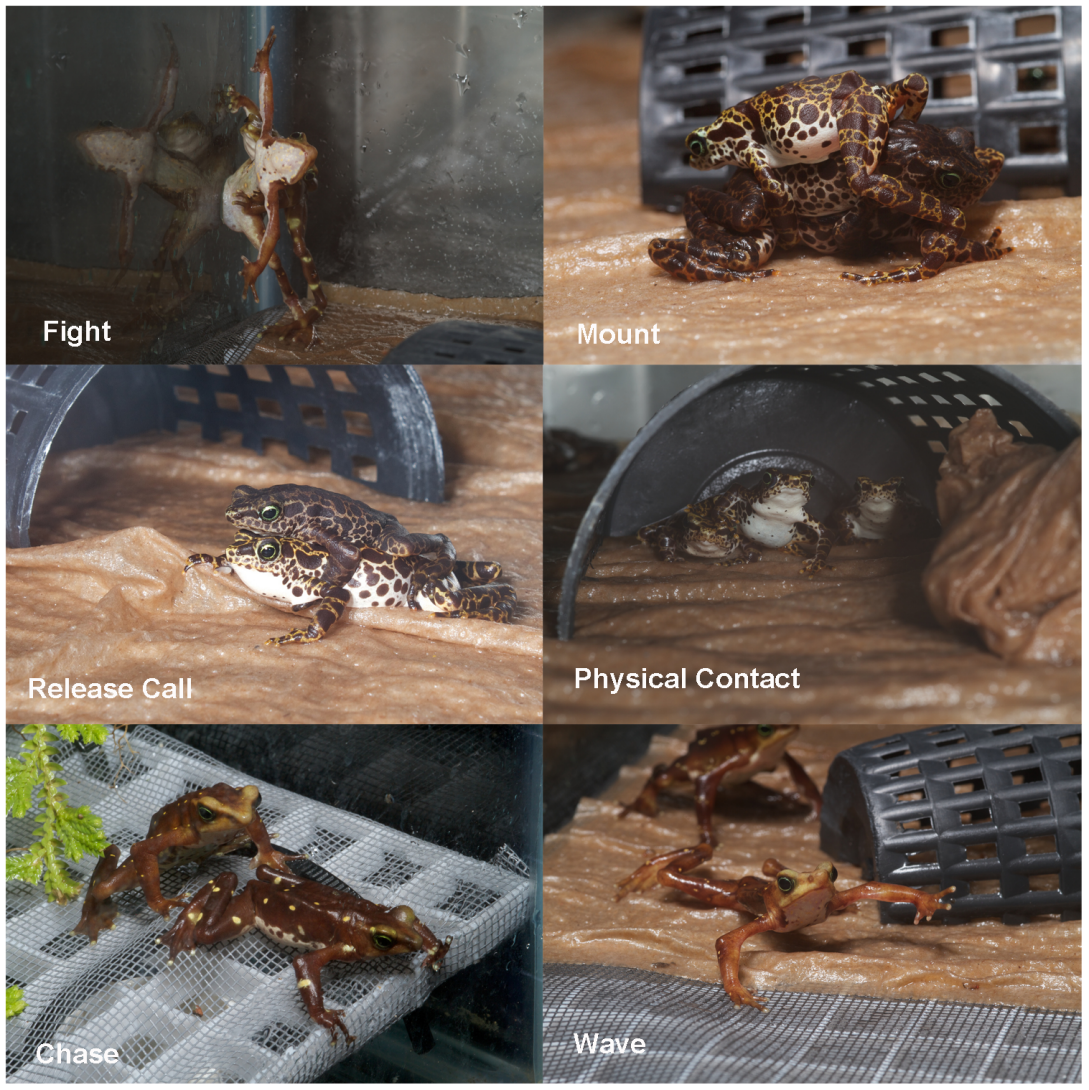
Ethogram describing different types of aggressive interactions observed for *Atelopus*. **Fight**: Combat involving mouth or front limbs, often flipping of opponent; **Mount**: >50% of initiators body covers the victim for >5 seconds; **Release call**: High pitched, weak, peep like call; maximum tally of one per individual; **Physical contact**: Any remaining forms of physical contact; **Stalk**: One individual actively follows/chases another for >5 seconds; **Wave**: Circular movements in front limbs.

A range of territorial and aggressive behaviors were recorded to assess the degree of conflict associated with each group size and defined using an ethogram (Figure 1). Aggressive interactions included fighting, mounting, release-call, stalking, and waving. A single observer (SN) tallied behavior in each tank for 5 minutes twice a day, in the morning between 0700–0830 hr and in the afternoon between 1400–1530 hr. The order of sampling was randomized to prevent any sequential bias due to time of day. All observations in a single week were summed and divided by the number of frogs in each tank to obtain a total number of aggressive interactions observed per frog per week.

### Fecal collection

Fecal material was removed manually and tanks were not changed for the duration of the experiment. Each week a single frog produces 5–9 fecal pellets with a mean weight of 0.038 g SD+/−0.026 g. Fecal pellets were stored at −20°C until extraction and analysis of GC metabolite concentrations. Collection began 1 week prior to moving frogs to the glass cages (week 0) to establish baseline GC concentrations. Every solid fecal sample was collected within 12 hours of being voided and all weekly samples from each enclosure were frozen together in 1.5 mL polypropylene tubes until processing. Samples from each cage were pooled by week to obtain a sufficient weight of fecal material for extraction.

### Fecal glucocorticoid extraction and analysis

A fecal GC extraction method similar to Brown et al. [23], [24] was used for frog feces. Four solvent:water (v:v) ratios were tested to determine the best recovery: 90% ethanol:dH_2_O, 80% ethanol:dH_2_O, 90% methanol:dH_2_O and 80% methanol:dH_2_O. The subsequent extracts were serially diluted, analyzed on the cortisol EIA (described below), and compared to the standard curve for parallelism (90% ethanol: *r* ^2^  = 0.988, *F*(1,4)  = 316.64, *p*<0.01; 80% ethanol: *r* ^2^  = 0.980, *F*(1,4)  = 397.92, *p*<0.01, 90% methanol: *r* ^2^  = 0.995, *F*(1,5)  = 1093.27, *p*<0.01 and 80% methanol: *r* ^2^  = 0.995, *F*(1,4)  = 758.56, *p*<0.01). For each method, the linear portion of the slope of the curve was similar to the standard curve (standards: −11.74; 90% ethanol: −11.84; 80% ethanol: −11.78; 90% methanol: −10.98 and 80% methanol: −12.16). Although all solvent ratios resulted in high steroid recovery, based on the maximum percent binding (%B) of a neat sample, 90% methanol:10% dH_2_O was the optimal extraction method (90% ethanol, 41.36%B; 80% ethanol, 38.95%B; 90% methanol, 31.59%B; 80% methanol, 37.04%B). Thus, for this study, wet samples (∼0.05 g) were weighed and placed into 16×125 mm borosilicate tubes. Five mL of 90% methanol:10% dH_2_O was added to each sample, tubes were capped and then vortexed for 10 seconds. Samples were shaken on a large capacity mixer for 30 minutes (Glas-Col, Terre Haute, Indiana, speed 55, pulse rate 1/second) followed by centrifugation at 2500×g for 20 minutes. The supernatant was recovered, and 5 mL 90% methanol:10% dH_2_O was again added to each tube. The pellets were re-suspended and the samples were shaken on a large capacity mixer (30 seconds, speed 55, pulse rate 1/second) and centrifuged for 20 minutes. The supernatants were combined, evaporated to dryness under directed air, then reconstituted in 1 mL 100% methanol and placed in an ultrasonic cleaner water bath (Cole Parmer Instrument Company, Vernon Hills, Illinois) for 10 minutes and dried down. Fecal extracts were reconstituted in 1 mL preservative-free buffer (0.2 M NaH_2_PO_4_, 0.2 M Na_2_HPO_4_, 0.15 M NaCl; pH 7.0), sonicated for 15 minutes, transferred to polypropylene tubes and stored at −20°C until analysis. One hundred µL of ^3^H-cortisol (∼10,000 cpm/100 µL) was added to monitor extraction efficiency of each sample, which was determined to be 90±0.003% (mean ± SEM) based on recovery of radioactivity after extraction.

**Figure 2:**
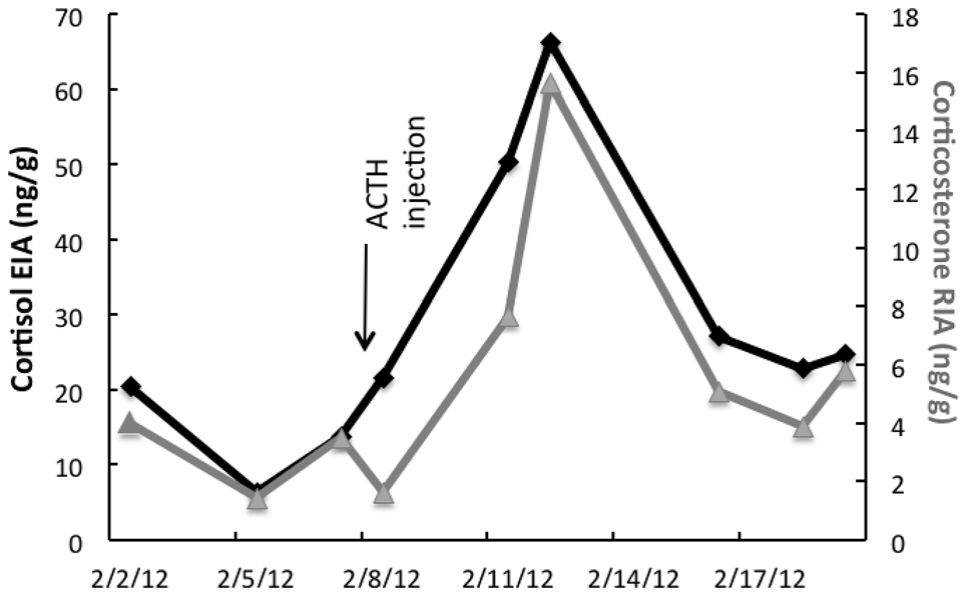
Cortisol EIA profile (black, diamond marks) and Corticosterone RIA profile (grey, triangle marks), in *Atelopus* feces following an ACTH challenge (0.2 IU, IM).

Two assays, a cortisol enzyme immunoassay (EIA; C. J. Munro, University of California, Davis, California) and a corticosterone radioimmunoassay (RIA; MP Biomedicals, Santa Ana, California), both of which have broad crossreactivity with fecal GC metabolites in numerous species [25], [26] were evaluated for use with Panamanian golden frog (*A. zeteki*) feces. Both the corticosterone RIA and cortisol EIA demonstrated parallelism between serial fecal extract dilutions and the respective standard curve. Low matrix interference was indicated in the corticosterone RIA as a result of 88% recovery of known standard concentrations when diluted with equal parts fecal extract pool. For the cortisol EIA, the average recovery was 91%. To compare longitudinal patterns, samples from 11 frogs were analyzed in both assays and the correlation between the two was calculated. The median correlation between the assays for individual fecal GC profiles was high at *r* = 0.92 (range: 0.57–0.98). Biological validity was shown by a frog that demonstrated a marked increase in GC concentrations within 4 days after ACTH injection (0.2 IU, IM) for both EIA (pre, 13.8 ng/g; post, 66.4 ng/g) and RIA (pre, 2.9 ng/g; post, 15.7 ng/g); the two profiles were correlated (*r* = 0.93; *p*<0.001) (Figure 2). Thus, both assays were able to detect similar patterns of hormone excretion; however, the cortisol assay detected higher overall concentrations of metabolites (∼5-fold higher) and so was used in this study.

The single-antibody cortisol EIA was based on the methodology of Munro and Lasley [27] and used a polyclonal antiserum (R4866) and horseradish peroxidase ligand (lot 051229, SCBI, Front Royal, Virginia). The cross-reactivities for R4866 are: cortisol 100.0%, prednisolone 9.9%, prednisone 6.3%, cortisone 5.0%; all other compounds cross-react with the antibody <1.0% [26]. The standard curve range for the assay was 0.78–20.00 ng/mL. Antiserum was diluted with coating buffer (0.015 M Na_2_CO_3_, 0.035 M NaHCO_3_, pH 9.6) and adsorbed to NUNC Maxi-sorp flat-bottomed, 96-well microtiter plates overnight at 4°C. The plate was washed 5 times (0.05% Tween 20 in 0.15 M NaCl solution), then 50 µL of standards, internal controls and samples were loaded onto the plate in duplicate, followed by the addition of 50 µL diluted horseradish peroxidase solution. Assays were incubated at room temperature for 1 hour, washed 5 times and 100 µL of 2,2′-azinobis [3-ethylbenzothiazoline-6-sulfonic acid]-diammonium salt [ABTS] solution (0.04 M ABTS, 0.5 M H_2_O_2_ in 0.05 M citric acid buffer) added to every well. Absorbance was read on a spectrophotometer (MRX, Dynex Technologies, Chantilly, VA, with a 405 nm filter 405 and reference filter of 490 nm) until the optical density (OD) of the 0.00 ng/mL standard reached ∼1.0 (range: 0.9–1.1; desired OD reached within 20–30 min). Steroid concentrations were divided by the amount extracted and reported as ng/g feces. Samples weighing <0.01 g were excluded from the data set because low weight samples consistently exhibited higher values compared to heavier samples [28]. The inter-assay variation on two internal controls (high and low GC concentration) were 7.3 and 8.0% CV, respectively (n = 16). Intra-assay variation between sample duplicates was <10% CV.

### High performance liquid chromatography

High performance liquid chromatography (HPLC; Varian ProStar; Varian Analytical Instruments, Lexington, Massachusetts) was used to characterize the numbers and proportions of immunoactive hormone metabolites excreted in *Atelopus* feces. Three aliquots of pooled fecal samples were extracted as described above, omitting the ^3^H tracer. The methanol extracts were pooled, dried down under directed air, resuspended in 500 µL PBS (0.03 M Na_2_HPO_4_, 0.02 M NaH_2_PO_4_, 0.15 M NaCl, 0.002 M NaN_3_, pH: 5.0), filtered through a C18 Spice cartridge and evaporated to dryness. For chromatographic markers, approximately 14,000 cpm/mL of ^3^H-cortisol and ^3^H-corticosterone were each added to the extract. The extract was dried down then reconstituted in 300 µL methanol (HPLC Grade Methanol, Fisher Scientific, Pittsburgh, Pennysylvania) and sonicated for 15 minutes. Then, 50 µL of extract was loaded onto a reverse-phase C18 HPLC column (Agilent Technologies, Santa Clara, California) with a 20–80% linear gradient of HPLC Grade methanol:water over 80 minutes (1 mL/minute. flow rate, 1 mL fractions). A 50 µL aliquot of each fraction was analyzed for radioactivty using a multi-purpose β-radiation scintillation counter (LS 6500, Beckman Coulter, Brea, California). The remaining volume was dried down, reconstituted in 200 µL preservative-free phosphate buffer and analyzed in singlet in the cortisol EIA and corticosterone RIA.

### Statistical analysis

Data were analyzed using SYSTAT 11 software. We performed a repeated measures ANOVA using GC values (ng/g) or feces extracted from pooled weekly fecal samples collected in each tank as the repeated measure, and group size (two frogs per tank vs. eight frogs per tank) was our experimental factor. For the group size of n = 1, one week's worth of fecal pellets was sometimes not enough material to perform an extraction, resulting in too many missing values to be incorporated into the statistical analysis. For the behavioral analysis, we performed repeated measures ANOVA using total number of aggressive interactions observed per frog per week as the repeated measure and group size (two frogs per tank vs eight frogs per tank) as our experimental factor.

## Results

### High performance liquid chromatography

Based on HPLC analysis, ^3^H-cortisol eluted at fractions 39–41 and peaked at fraction 40, while peak ^3^H-corticosterone eluted at fraction 45 (range: 44–46). Immunoactivity of fractions analyzed on the cortisol EIA indicated the presence of native cortisol with a peak at fraction 39 (15% of the total immunoactivity) and a smaller amount of immunoreactivity at fractions 44–45 (11%). Additional immunoreactivity was observed at fraction 13 (6%), and there were peaks of uncharacterized less polar metabolites at fractions 54, 59, 66, 75, and 79 (68% of total immunoactivity). Concentration of GC immunoactivity of HPLC-separated fractions in the corticosterone RIA was only about a tenth that of the cortisol EIA, with small peaks at 37-39 (3%) and 44–48 (11%), and also several less polar peaks of similar levels of immunoreactivity at fractions 50, 54, 59, 63, 65, 76, and 79 (86%).

### Behavioral responses

**Figure 3:**
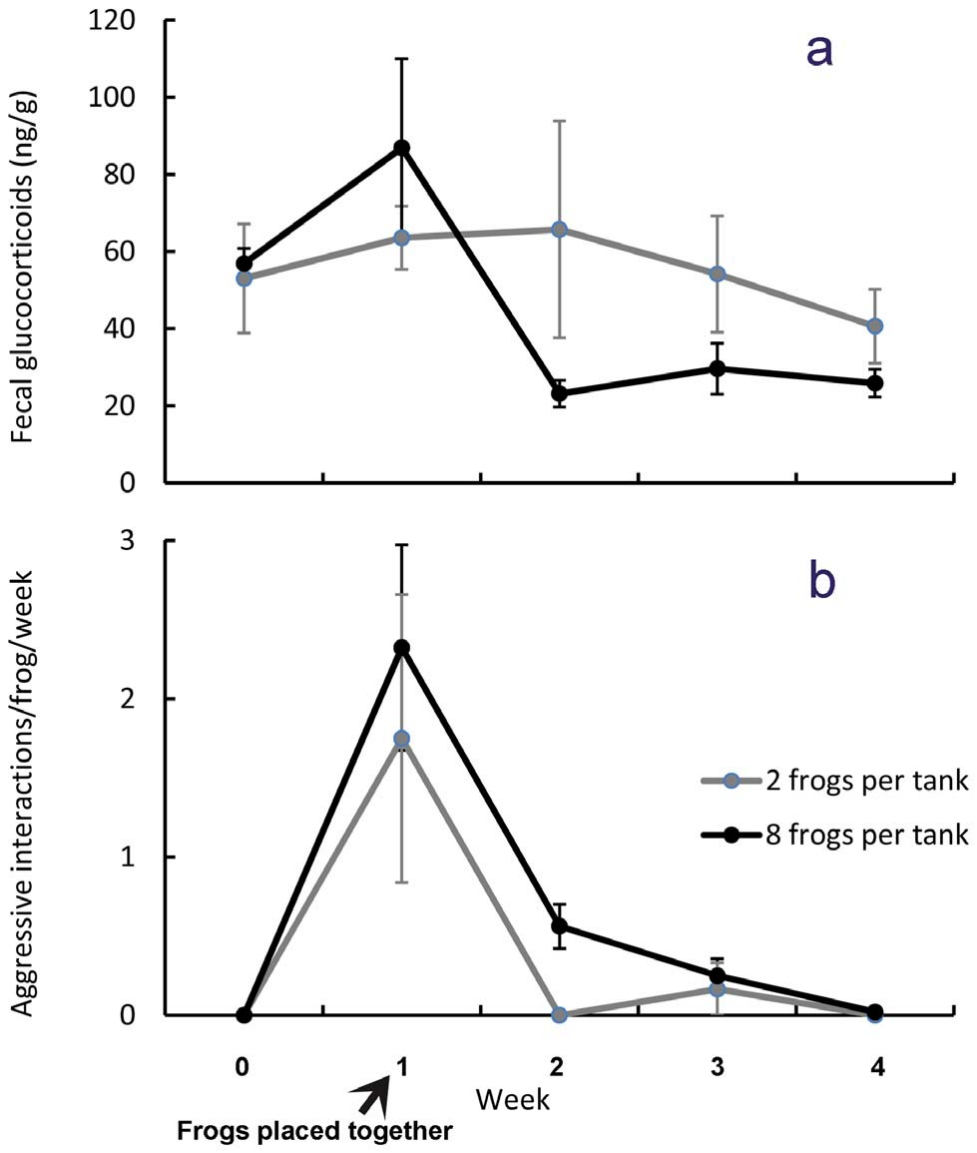
Fecal glucocorticoid concentrations immediately before and after male *Atelopus* were grouped together at week 1 (ng cortisol/g ± SEM) changed significantly over time (p = 0.04*), but there were no significant differences between groups sizes (A). Frogs housed singly (mean  = 44.2 ng cortisol/g±7.4 SEM) could not be included in this analysis because of too many missing values. Aggressive interactions changed significantly over time (p<0.001***), but there were no significant differences between group sizes.

The most common behavior observed was physical contact, which accounted for 28% of all aggressive interactions included in the ethogram, but for the purposes of this analysis, all aggressive interactions were pooled because no single behavioral response was recorded in high enough frequency to compare behavior types. When we placed groups of both two and eight *Atelopus* together, aggressive interactions were high at first, but declined significantly in subsequent weeks to a mean of almost zero aggressive interactions observed per frog per week (Figure 3B, Table 1B). There was no significant difference in relative number of aggressive interactions per frog, between groups of 8 and 2 frogs.

**Table 1:** Repeated measures ANOVA testing the effects of time (weeks 0–4) and group size on fecal glucocorticoid levels in *Atelopus* housed together (groups of 8 vs 2).

a Fecal Glucocorticoids	F	df	P
Group size	1.217	1	0.296 NS
Week	2.768	4	0.04*
Week*Group size	1.728	4	0.163 NS
**b Aggressive Interactions**			
Group size	1.128	1	0.313 NS
Week	11.009	3	<0.001***
Week*Group size	0.278	3	0.841 NS

*(* ***a*** *). We omitted group size n = 1 from the analysis because there were too many missing values to run statistical comparisons. Repeated measures ANOVA testing the effects of time (weeks 1–4) and group size (8 vs 2) on aggressive interactions in Atelopus housed together (* ***b*** *).*

### Physiological responses

Similarly, there was a significant increase in fecal GC levels in groups of both two and eight frogs per tank when they were placed together for the first time. Fecal GC concentrations in groups of two and eight individuals rose initially and then declined to their lowest concentrations by week 4 (Figure 3A, Table 1A) when mean fecal glucocorticoid levels approximated the mean glucocorticoid levels of individually housed frogs 44.2±7.4 ng/g. Interestingly, the cortisol levels by the end of the experiment were both lower than the baseline (week  = 0) levels obtained when they were being housed individually in small plastic cages. Overall body condition measurements had a high degree of variability, but on average frogs lost 2.6% of their body mass over the 5 weeks SD +/−7.1%. There were no statistically significant differences in changes in body condition between frogs in the different group sizes (Kruskall-Wallace p 0.454).

## Discussion

### Fecal glucocorticoid extraction methods

In comparative analyses, a cortisol EIA and corticosterone RIA were both capable of quantifying GC metabolites in *Atelopus* fecal extracts, and results were highly correlated. The cortisol antibody reacted with metabolites that co-eluted with both tritiated cortisol and corticosterone tracers, and also a number of less polar metabolites. The corticosterone RIA appeared to detect a small amount of native corticosterone, but also significant amounts of less polar metabolites not unlike that observed for the EIA, although at much lower levels. We decided to employ the cortisol EIA because it detected more immunoreactive mass, it is more portable for possible field studies, and does not rely on the use of radioactive tracers.

### Behavioral and physiological responses to grouping

We demonstrated that wild-caught male *Atelopus* do interact aggressively when housed in both small and large groups, but over a relatively short period of time these frogs become accustomed to each other and reduce the frequency of their aggressive interactions. A similar pattern was observed in fecal GC concentrations, indicating that these aggressive interactions are likely associated with a physiological stress response. Interestingly, the mean baseline GC values pre-grouping were higher (60 ng/g) than might have been expected based on the mean GC values observed at the conclusion of the experiment (40 ng/g; Figure 3A). This may be connected to the fact that, prior to the group housing, the smaller holding cages were completely changed twice per week, involving frequent handling of the frog that may have slightly elevated GCs. Once the frogs were transferred to their larger glass cages with automated misting and draining systems we did not need to handle the frogs at all for the duration of the experiment. Many other studies have examined GC levels in amphibians; e.g., [17], [18], [20], [21], but this is the first time that fecal GCs have been used to evaluate stress in frogs. Our findings are similar to observations by others that group housing of cane toads led to increased urinary corticosterone concentrations that declined once they were moved to individual housing [21]. Measurements of GCs using other methods such urine sampling or buccal swabs can potentially give accurate, short-term measurements e.g. [20]. However, the frogs in this study were not used to handling, so invasive procedures like blood and buccal sample collection would have in themselves induced stress. These approaches would have permitted more fine-scale measurements of acute stress responses, but this was not our objective. Urine is an effective noninvasive approach, but housing conditions of the frogs in our study did not permit reliable sample collection. We also could not partition stress by individual frogs to see if some individuals were experiencing more or less stress than others. But the one major advantage to feces is that it represents a pooled sample over time, so acute fluctuations among and between individuals are dampened. Thus, we conclude that fecal steroid monitoring provides a minimally invasive option to researchers that can be applied, as we have demonstrated here, to evaluate and compare husbandry practices, and how they impact frog welfare over time, as efforts to build global amphibian arks for endangered amphibians continue to grow [28].

Our results are significant from a conservation perspective because they justify housing frogs in groups. Given that space is a major limiting factor in *ex-situ* conservation programs, it will greatly increase the number of frogs that can be held in captivity and managed for amphibian conservation and reintroduction efforts. We did not detect a statistically significant effect of group size; this may have been due to low statistical power from having just six replicates. Future studies that increase the number of replicates and treatment groups may shed light on optimal housing densities. It is important to note, however, that housing frogs in groups may have other consequences not addressed in this study. For example, group housing may lead to changes in body condition if smaller or non-dominant animals do not compete as well for food [29], but this was not observed in this experiment. Group housing may lead to increased buildup of gut parasite loads [29] or increased aggressive interactions during the breeding season [11]. Any of these could have an impact on the long-term health of an individual if not carefully managed and monitored by animal care staff, and should be considered carefully before making management changes.

## Supporting Information

Table S1 **Summary data of fecal glucocorticoids, aggressive interactions, weight change, grouping information, and individual frog accession numbers.**
(XLS)
